# Tuberculous mastitis simulating carcinoma of the breast in a young Nigerian woman: a case report

**DOI:** 10.11604/pamj.2015.21.125.6336

**Published:** 2015-06-15

**Authors:** Donatus Sabageh, Emmanuel Afolabi Amao, Adebisi Ayo-Aderibigbe A, Adedayo Olukemi Sabageh

**Affiliations:** 1 Department of Morbid Anatomy and Histopathology, Ladoke Akintola University of Technology Teaching Hospital, Ogbomoso, Oyo State, Nigeria; 2 Shalom Medical Centre, Ogbomoso, Oyo State, Nigeria; 3 Department of Community Medicine, Ladoke Akintola University of Technology, Ogbomoso, Oyo State, Nigeria

**Keywords:** Tuberculous mastitis, mimic, breast carcinoma, Nigerian woman

## Abstract

Tuberculous mastitis is an uncommon disease even in countries where tuberculosis is highly endemic. It typically presents a diagnostic challenge masquerading as carcinoma or other primary disease of the breast. We report the case of a young multiparous Nigerian woman who presented with a tender left breast lump and enlargement of the left axillary lymph nodes for which a provisional diagnosis of carcinoma of the breast was made after clinical and radiological evaluation. The mass was pathologically diagnosed as tuberculous mastitis and anti-tuberculous therapy was instituted although she later absconded. This case shows that TM may present a diagnostic challenge on clinical, radiologic and microbiological investigation. Therefore, a high index of suspicion as well as FNAC and/or histological evaluation of tissue samples remain very important its diagnosis.

## Introduction

Tuberculous mastitis (TM) is an uncommon disease even in countries where the incidence of pulmonary and extrapulmonary tuberculosis is high [1, 2]. The incidence is known to range from 0.1% in developed countries to about 4% in highly endemic countries like India [3, 4]. The incidence in Nigeria is however unknown due to the paucity of information about the disease [5]. TM remains a diagnostic dilemma with several recent reports showing TM masquerading as carcinoma or other primary disease of the breast [5]. It typically affects young lactating multiparous women and can present as either an abscess or as a unilateral, painless breast mass [6]. TM is paucibacillary and consequently, histological and/or cytological evaluation of diseased breast tissue remains crucial to the diagnosis [6]. Here we report the case of TM in a young multiparous and lactating Nigerian woman which was clinically misdiagnosed as carcinoma of the breast.

## Patient and observation

**Figure 1:**
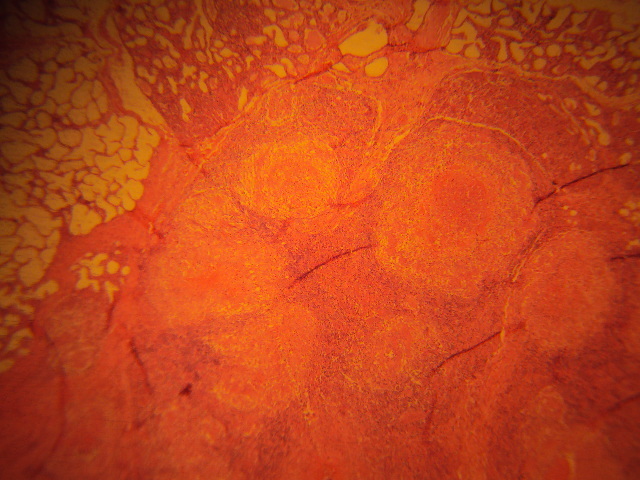
Low power view showing a few granulomas with central caseous necrosis and thin peripheral rim of lymphocytes. Normal breast glands showing pregnancy-associated changes can be seen at the upper edges of the photomicrograph (H&E, X80)

**Figure 2:**
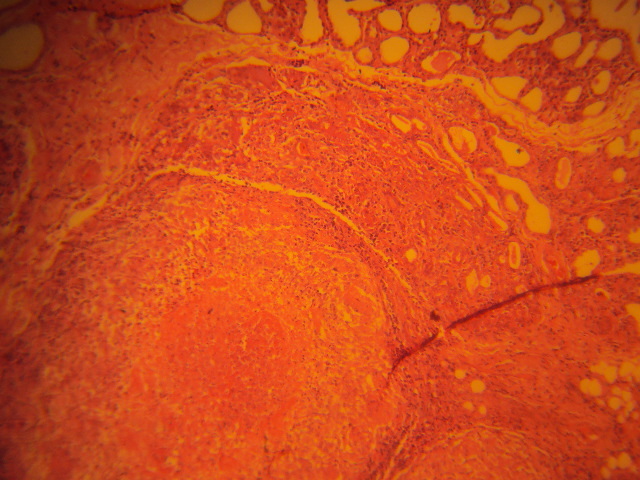
Higher power view shows one of the granulomas with an obvious central area of caseous necrosis surrounded by epithelioid cells among which are seen a few multinucleated giant cells. Normal breast glands with pregnancy-like changes are seen at the upper end of the micrograph (H&E, X200)

A 29-year old multiparous nursing mother presented to a private hospital in Nigeria with a week history of pain in the left breast which was associated with severe itching sensation. There was no nipple discharge. She denied any history of fever, night sweats or weight loss. She also had no family history of breast cancer. She had been on some antibiotics and pain relievers before presentation at the hospital. General physical examination was normal. The breasts were asymmetrical with the left breast bigger than the right. Two discrete and tender masses were palpable in the upper outer quadrant of the left breast. These measured 4.0cm x 3.0cm and 3.0cm x 2.0cm respectively. They were mobile and not attached to the overlying skin. The nipple, areola and overlying skin were normal. Three discrete, non tender left axillary lymph nodes were also palpable. The right breast was, however, normal. A provisional diagnosis of carcinoma of the left breast was made based on the clinical findings. This was further buttressed by the ultrasound scan performed on the left breast. A mammography was requested for but this could not be done for technical reasons. Her chest X-ray was normal while her blood smear did not show any microfilariae. A complete blood picture showed a total white cell count of 0.7 x 10^9^/L (normal 4-11 x 10^9^/). An excision biopsy was thereafter performed and sent for pathological examination. This revealed an unencapsulated, irregularly shaped fibrofatty soft tissue measuring 7.0x6.0x4.5cm. Histology, however, showed numerous granulomas with central caseous necrosis surrounded by typical epithelioid macrophages and a thin rim of lymphocytes (Figure 1). There were a few Langhans type multinucleated giant cells in between the macrophages (Figure 2). The surrounding breast lobules were expanded and showed pregnancy-like changes. The histopathological diagnosis was tuberculous mastitis even though the Ziehl Neelsen stain performed on the tissue sections was negative. A Mantoux test was done and this showed a positive reaction after 48 hours. She was commenced on a 6-month anti-tuberculous therapy (isoniazid, rifampicin, pyrazinamide and ethambutol) but she defaulted and was lost to follow up.

## Discussion

Tuberculous mastitis (TM) is a rare disease all over the world and its incidence rate is known to be higher in countries where tuberculosis is endemic [1–4]. Nevertheless, there has been a recent global increase in the incidence rates owing to the resurgence of pulmonary and extrapulmonary tuberculosis developing in the background of the human immunodeficiency virus scourge [3, 7, 8]. The incidence rates in Nigeria are unknown due to the paucity of information about TM from Nigeria [5]. The general rarity of TM is attributable to the remarkable resistance offered by the breast tissue to the survival of the causative organism [9]. In secondary TM, however, infection may spread to the breast from other affected organs which may not be apparent clinically or by means laboratory or radiological investigation [9]. In these cases, the major routes of spread are lymphatic, haematogenous and contiguous spread from the pleura and chest wall. Lymphatic spread, which is the commonest, is typically by retrograde extension from axillary nodes or occasionally cervical and mediastinal nodes [4, 8, 10]. Such cases typically present with a breast mass in the upper outer quadrant of the breast associated with axillary lymph node enlargement. This was the mode of presentation in our patient. This mode of presentation may be indistinguishable from carcinoma of the breast since the breast masses are typically hard, irregular, ill defined, and may be fixed to either the skin or chest wall [2]. However, the breast masses in TM are usually painful and solitary [8]. The index case presented with two tender masses. The diagnostic confusion this poses may be further compounded by radiological tools like mammography and ultrasonography which are generally useful only in defining the extent of the lesion but not in differentiating it from other diagnostic possibilities, especially carcinoma of the breast with which it shares similar characteristics [2]. Patients with TM less commonly present with a tuberculous ulcer over the breast skin or a tuberculous abscess with or without discharging sinuses especially in younger patients. Peau d'orange is often seen in patients with extensive axillary nodal tuberculosis [8, 10]. The presence of constitutional symptoms and the lack of involvement of the nipple and areola are important in distinguishing TM from carcinoma. Both breasts are rarely involved together in TM [5, 11]. Although TM is reported to affect females within the reproductive age group of 21-40 years most commonly, the associated risk factors generally include multiparity, lactation, trauma, past history of suppurative mastitis and AIDS [5]. Primary TM, though much less common than the secondary form, may occur through abrasions in the breast skin or through the duct openings on the nipples [4]. Our patient was a multiparous and lactating woman within the reproductive age group. The increased incidence of TM in pregnancy and lactation may be attributable to the increased vascularity and dilation of breast ducts that predispose them to trauma and therefore tuberculous infection [4, 5]. TM is extremely rare in males, prepuberscent females and elderly women [4, 7].

It is important that TM be distinguished from other granulomatous diseases of the breast including sarcoidosis, fungal infections, foreign body reactions, mammary duct ectasia, traumatic fat necrosis among others [4, 6]. Histopathology and fine needle aspiration cytology (FNAC) remain the main tools for the accurate diagnosis of TM, failure to demonstrate acid fast bacilli notwithstanding [4]. It is known that the demonstration of acid fast bacilli (AFB) from the lesions is usually difficult with bacilli being isolated in only 25-30% of cases and AFB identified in only about 12% of patients [9, 11]. The presence of either caseating granulomas with Langhan's giant cells from the breast tissue and involved lymph nodes may therefore be sufficient [4]. These features were clearly demonstrated in the index case. The role of nucleic acid amplification tests is debatable especially in AFB smear negative cases as they suffer from low sensitivity and other limiting factors [2, 6]. Three patterns are apparent on histological evaluation namely, nodular, diffuse and sclerosing [4, 5, 7]. The nodular variety, as seen in the index case, is often mistaken for a fibroadenoma or carcinoma. The diffuse type commonly leads to sinus tract formation while the sclerosing variety is slow growing and typically afflicts older women [5, 7]. It is recommended that TM be treated as any other form of extrapulmonary tuberculosis generally for 6 or 9 months unless drug resistance is present [5]. Success rates following medical therapy approaches 95% in most series [2]. Surgical intervention may, however, be needed in up to 14% of patients especially when medical therapy fails, large painful ulcers develop or for the drainage of cold abscesses in the breast or axilla. Simple mastectomy is rarely needed [2].

## Conclusion

Tuberculous mastitis is uncommon even in countries where tuberculosis is highly endemic. It can present a diagnostic challenge on clinical, radiologic and microbiological investigation. Therefore, a high index of suspicion as well as FNAC and/or histological evaluation of tissue samples remain very important its diagnosis.
